# Place Field Repetition and Purely Local Remapping in a Multicompartment Environment

**DOI:** 10.1093/cercor/bht198

**Published:** 2013-08-13

**Authors:** Hugo J. Spiers, Robin M. A. Hayman, Aleksandar Jovalekic, Elizabeth Marozzi, Kathryn J. Jeffery

**Affiliations:** 1 Department of Cognitive, Perceptual and Brain Sciences, Division of Psychology and Language Sciences, Institute of Behavioural Neuroscience, University College London, UK; 2 Axona Ltd, Unit 4U St Albans Enterprise Centre, St Albans, UK

**Keywords:** grid cells, path integration, place cells, rat, spatial memory

## Abstract

Hippocampal place cells support spatial memory using sensory information from the environment and self-motion information to localize their firing fields. Currently, there is disagreement about whether CA1 place cells can use pure self-motion information to disambiguate different compartments in environments containing multiple visually identical compartments. Some studies report that place cells can disambiguate different compartments, while others report that they do not. Furthermore, while numerous studies have examined remapping, there has been little examination of remapping in different subregions of a single environment. Is remapping purely local or do place fields in neighboring, unaffected, regions detect the change? We recorded place cells as rats foraged across a 4-compartment environment and report 3 new findings. First, we find that, unlike studies in which rats foraged in 2 compartments, place fields showed a high degree of spatial repetition with a slight degree of rate-based discrimination. Second, this repetition does not diminish with extended experience. Third, remapping was found to be purely local for both geometric change and contextual change. Our results reveal the limited capacity of the path integrator to drive pattern separation in hippocampal representations, and suggest that doorways may play a privileged role in segmenting the neural representation of space.

## Introduction

One way to distinguish identical parts of the environment, such as identical offices along a corridor, is to use their relative spatial locations. This knowledge could, in principle, be informed by path integration, the self-motion-tracking process that updates the internal representation of position independently of landmarks (Mittlestadt and Mittlestadt, 1982; Etienne and Jeffery 2004; McNaughton et al., 2006). Self-motion information can be derived from optic flow registered on the visual system, but also from processing information about the translation and rotation of the body in space (Etienne and Jeffery 2004). The hippocampus is thought to integrate path integration inputs with information about visual landmarks and boundaries to form an internal map of the environment (O'Keefe and Nadel, 1978). Cells within the hippocampus, known as “place cells’,” show location specific firing, with each cell responding in a region termed the cell's “place field.” The present study explored whether the hippocampal place cell system, can use path integration to disambiguate visually identical subregions of an environment.

Evidence indicates that place cells use both landmark-based and path integration information to locally position their place fields (Gothard et al. 1996; Etienne and Jeffery 2004; McNaughton et al. 2006), and early studies suggested they can use path integration information to distinguish identical compartments in a 2-compartment chamber (Skaggs and McNaughton 1998; Tanila 1999). When lights are extinguished during exploration of an environment place fields have been found remain stable, at least during the initial part of the trial (O'Keefe, 1976; Quirk et al. 1990; cf. Save et al. 2000). The neural origin of path integration received by place cells is speculated to arise from grid cells in parahippocampal structures (Hafting et al. 2005; McNaughton et al. 2006; Boccara et al., 2010), whose regular, repeating firing fields indicate the operation of a self-motion-based process. Although it is still to be established whether grid cells provide the main source of path integration to place cells, the properties of grid cells can explain a number of the self-motion-related properties of place cells (McNaughton et al. 2006; Moser et al. 2008). Thus, if place cells can use path integration to generate separate representations of 2 compartments, it is plausible that place cells and grid cells would form separate representations of compartments in environments containing more than 2 compartments. However, Derdikman et al. (2009) showed that in fact, place cells and grid cells recorded in an environment comprising multiple identical tracks actually repeat their firing fields across tracks, with grid fields being “reset” each time the animal entered a new track and therefore not discriminating compartments. This finding seems inconsistent with the findings in place cells of Skaggs and McNaughton (1998), who observed considerable discrimination between compartments, as did subsequent studies of “path equivalence” in repeating linear environments (Singer et al. 2010). However, studies finding discrimination have used binary environments and/or environments with perceptible extramaze cues, while studies showing path equivalence have used environments in which animals ballistically execute highly stereotypical movements. It remains unknown whether in an ordinary foraging situation, in which animals can re-enter multiple compartments at will, place cells can use pure path integration to keep the representations of identical compartments separate.

**Figure 1.**
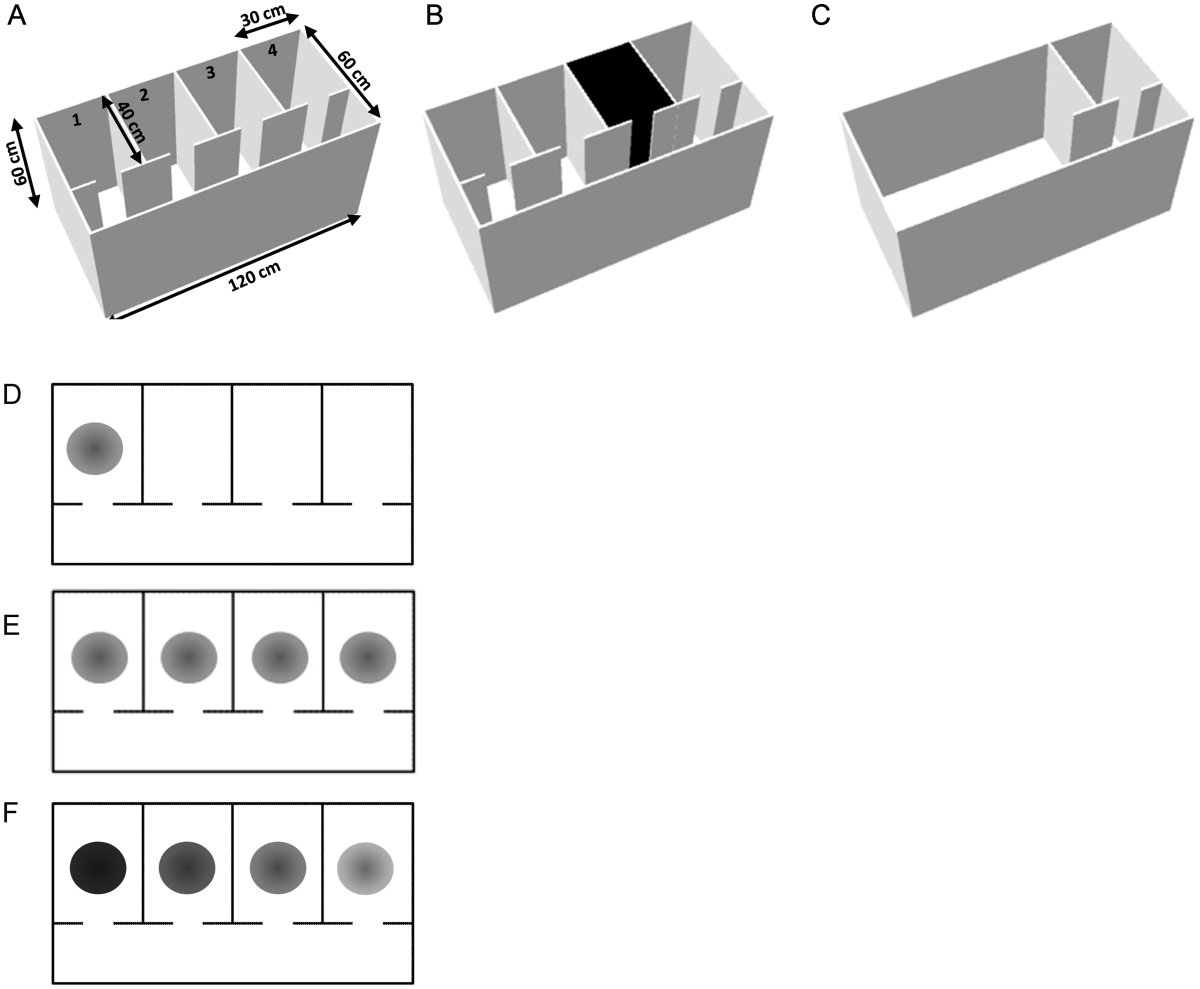
(*A–C*) Schematic of the apparatus in its 3 configurations: (*A*) The standard configuration showing 4 main identical compartments, connected by a long corridor. (*B*) The context remapping manipulation, in which 1 of the 2 middle compartments was changed from white to black by adding wall and floor inserts. (*C*) The wall-removal manipulation in which all of the interior walls except those surrounding the end compartment were removed. (*D–E*) Possible outcomes predicted on a plan view of the apparatus. Filled circles represent place fields from a single hypothetical place cell. (*D*) Spatial discrimination: If place cells are able to discriminate the compartments cells should produce unique firing patterns in the environment. In this example, a single field is shown, but other examples could include fields in each of the compartments, each occupying a different location. (*E*) No discrimination—place fields repeat across compartments, firing in the same location in each compartment. (*F*) Rate discrimination—place field locations repeat across compartments, but the peak rate is modulated across compartments. In this example, the highest peak rate (darkest circle) is in the first compartment, but the peak might occur in any of the compartments.

Accordingly, we revisited the repeating-compartment experiment with place cells, using an environment with 4 compartments, instead of only 2, in which rats would forage rather than run ballistically (Fig. 1*A*). We expected that if the path integration informs place cells of compartment identity, the place fields should discriminate between compartments (Fig. 1*D*). Conversely, if the path integrator “resets” anew in every compartment, then place fields should have the same location in each compartment (Fig. 1*E*). In some experiments place cells have been found to discriminate different contexts by differences in their firing rate (rate coding), while maintaining their field location (Hayman et al. 2003; Leutgeb et al. 2005). Thus, place cells might be predicted to express a rate-based discrimination across compartments (Fig. 1*F*). As we show, place cells repeated their firing locations across these 4 compartments, even after extensive experience, and demonstrated some degree of rate-based discrimination. In addition, there was a prominent clustering of activity around the doorways of the compartments, suggesting that doorways are a particularly salient environmental feature. We then tested whether firing patterns provide a global or local code of the environment by examining whether changes to parts of the environment altered firing in connected but more distant regions. Purely local firing alterations were observed. Thus, in a free foraging situation, the location of place fields appears to be determined not by path integration but by purely local factors. We suggest that this may be due to resetting of the grid fields as the animals pass through doorways.

## Materials and Methods

### Subjects

Ten adult male Lister Hooded rats (weighing between 270 and 470 g at the time of surgery) were housed individually [11:11 light:dark, with 1 h (×2) simulated dawn/dusk] on a food-restricted diet sufficient to maintain 90% of free-feeding weight, with ad libitum access to water. All procedures were licensed by the UK Home Office subject to the restrictions and provisions contained in the Animals (Scientific Procedures) Act 1986.

### Apparatus

The experiment was carried out in an environment with 60-cm high walls constructed from medium fiber density (MDF) boards and a white Plexiglas floor. The environment contained 4 identical compartments, labeled consecutively 1–4, and each 40 cm long, 30 cm wide, connected by a corridor 120 cm long and 20 cm wide (Fig. 1*A*). Each compartment was separated from the connecting corridor by 2 narrow walls, positioned to allow a 10 cm gap through which the rat could enter and exit the compartment. The outer walls and the 3 long dividers creating the 4 chambers were attached with corner clamps. The inner narrow walls used to create the door slits were attached with screws and brackets. For the context-change remapping manipulation (Fig. 1*B*), black color and altered odor were added by creating a thin insert lining composed of back polyurethane walls, and a black sandpaper floor. For the wall-removal condition (Fig. 1*C*), all walls except those enclosing the rightmost chamber were removed. The entire environment was surrounded by white curtains to conceal any room cues, and 2 lamps behind the curtains, in the north east and south east, provided diffuse light sources. The position of the rat was camera-monitored from overhead. Owing to the physical size of the environment and the presence of walls that partially obscured each compartment, it was not possible to capture the position of an animal using a single camera. To overcome this we used 2 cameras, where 1 camera was located above the common wall for compartments 1 and 2 and the other camera above the common wall for compartments 3 and 4. The corridor is shown in rate maps, but it was excluded from the analysis, and only data from within the compartments themselves were analyzed.

### Surgery and Electrodes

All rats were implanted at the start of the experiment with moveable microelectrodes. Four tetrodes were constructed from 4 interwound 25–µm diameter platinum-iridium wire (California Fine Wire, USA). The tetrodes were held in a microdrive assembly (Axona Ltd, St Albans, UK) that allowed them to be lowered or raised with one full turn of the screw equal to an increment of 200 µm dorso-ventrally. For 2 rats microdrives were implanted in both left and right hemispheres, for all other rats the implant was the right hemisphere. The animals were premedicated with buprenorphine and anesthetized with isoflurane and oxygen (3 L/min) before being placed on a stereotaxic frame, with lambda and bregma in the horizontal plane. Microdrives were fixed to the skull with 6 1.6-mm jewelers' screws (Precision Technology Supplies Ltd) and dental cement. One of the screws was soldered to a ground wire to enable the animal to be electrically grounded. The electrodes were lowered into the neocortex above the dorsal CA1 region of the hippocampus. Once the electrodes were implanted, a metallic sleeve was pulled down over the remaining exposed wires. Postsurgery the animals were monitored periodically until they awoke. All animals were given at least 1 week to recover following the surgery and treated with the analgesic for 3 days.

### Screening and Recording Procedures

Screening for place cells commenced 1 week after surgery, and took place in an area beyond the curtained environment in the same room, to minimize the learning of extraneous cues in the recording environment by the rats. Recording of single neuron activity was done using multichannel recording equipment (DacqUSB, Axona Ltd). The rats were connected to the recording device via lightweight wires and a socket attached to the microdrive plug. The potentials recorded on each of the 16 electrodes of the 4 tetrodes were passed through an AC-coupled, unity gain operational amplifiers, mounted on the rat's head and fed to the recording system. The signal was amplified (∼20 000 times), bandpass filtered (300 Hz–7 kHz) and then collected and stored on a computer. Each of the 4 wires of one tetrode was recorded differentially with respect to a wire from one of the other tetrodes. A headstage with 1 infrared LED array was used to track the rat's location. The video channels from the 2 ceiling-mounted cameras were first synchronized then mixed to create single video channel that alternated input from each camera at a rate of 25 Hz per camera. Once complex spikes were identified on the oscilloscope trace, the rats foraged on a 60 × 40 cm platform scattered with rice cooked with honey (which reduces chewing artifacts) while screening for place-specific unit activity was undertaken. If no place cell activity was present, the electrodes were lowered by ∼50 µm.

### Recording Procedures

Once place cells were found in a given rat, the animal was placed on a raised holding box outside of the curtained enclosure and connected to the recording device. After connection, the rat was placed facing into the corner of the corridor adjacent to Compartment 1. For every trial, the rat was allowed to forage throughout the whole environment for 15 min. Cooked rice grains were scattered throughout the trial so as to motivate the rat to explore every region of the environment evenly and without depleting any of the compartments.

Ten rats experienced at least one session with the maze in the standard arrangement (“standard” trials; see Fig. 1*A*), enabling assessment of multicompartment encoding in a novel environment. Eight rats underwent a second standard trial, with a 20 min delay between the trials, during which the rat was placed on the holding platform outside the curtained area with the recording headstage still connected. During the delay in these and all other trials, the inner walls of the environment were swapped to new positions to minimize the use of wall odor cues to discriminate the compartments. In between sessions the outer walls were also swapped and the whole floor rotated to a new orientation to re-configure the odor cues. In order to reduce the relative stability, and therefore influence, of room cues outside the curtained environment, between sessions the environment was pseudorandomly moved to 1 of 3 possible locations in the arena, each separated by 30 cm.

Two manipulations were then conducted with the environment: “context change” and “wall removal.” Five rats underwent the context-change manipulations, in which the lining of compartment 2 or 3 was changed by adding black plastic sheets to the walls and black sandpaper to the floor (Fig. 1*B*). The other 5 rats were not used due to insufficient cell yield (<3 complex spiking cells). All trials in each session were 15 min in duration. The delay between trials was 20 min during which the rat was placed on a holding platform outside the curtained area with the recording headstage still connected. For all rats, the first session involved 2 foraging trials with the maze in the standard configuration. For 5 rats on a subsequent day (mean = 9 days' delay), a standard trial was followed by a context-change trial.

Three of these rats, together with one newly implanted animal then took part in the geometric change condition manipulation. This was conducted in a separate session. For this manipulation, the environment change comprised removing some of the inner walls such that only compartment 4 remained (Fig. 1*C*), and the rest of the environment became an open field.

### Behavioral Analysis

The amount of time spent in each compartment was calculated by summing the amount of time spent in each bin of the positional maps. The dwell time in each compartment was then expressed as a percentage of the total time spent in all 4 compartments. Dwell times were calculated for the first-ever exposure to the multicompartment environment, the baseline trial preceding a context-change trial and the baseline trial following a context-change trial.

### Single Neuron Analysis

Cluster-cutting software (Tint, Axona Ltd) was used to analyze the data offline. For each tetrode, the peak-to-trough amplitude for each electrode was plotted against each other electrode, creating scatter plots in which spikes belonging to a single cell appeared as clusters. Manual cluster cutting was then used to separate these clusters.

The raw position data from the 2 cameras were integrated to create a composite map of the environment using custom scripts written in Matlab (MathWorks, USA). Experimenters manually merged the maps of the compartments by taking the 2 left-most maps from the left-hand camera and the 2 right-most maps from the right-hand camera, and scaling and aligning these by eye to make a single composite that showed all 4 compartments. The corridor, which was not of interest in this experiment, was excluded from the analysis. The position data were then smoothed using a moving average with a 400 ms boxcar, to smooth the path and remove noisy or mislocated points.

#### Firing Rate Maps

Firing rate maps were then generated as follows. For the between- and within-compartment correlation analysis, the extent of each of the compartments was defined as a rectangular area divided into an array of 9 × 12 square bins, each 3.3 × 3.3 cm in size. The total number of spikes found in a given bin was divided by the amount of time spent in that bin to provide a firing rate (Hz). Cells that fired over more than 75% of the total area of the 4 compartments or that did not reach a peak firing rate of 0.5 Hz in any of the compartments across the session, were excluded from further analysis. The remainder were considered putative place cells.

Smoothed firing rate maps were then generated using a spatial boxcar procedure in which the firing rate in each bin was replaced by that of the mean of itself plus the immediately surrounding bins (8 for central bins, 5 for edge bins and 3 for corner bins).

Correlations between the firing in different compartments, or the same compartment across different trials, were performed by comparing the place field map in each firing rate map to all other rate maps on a bin-by-bin basis. For any given comparison between 2 maps, areas devoid of sampling were removed from the correlation procedure to avoid artificially inflating the correlation values due to areas of common zeroes. Pairwise Pearson's Product Moment Correlation Coefficients were calculated between the bins in the 2 maps. This procedure was used to construct correlation matrices for all of the possible comparisons within a session. In order for cells to be entered into remapping analysis, a criterion mean correlation coefficient between the before and after baseline trials was set to be 0.40.

For the wall-removal trials, analysis was done by using imaginary boundaries corresponding to the dimensions and positions of the previous compartments, to define rectangular areas that allowed Pearson's correlation in the same way as before.

#### Autocorrelation Procedure

Given the spatial periodicity of the firing exhibited by many of the place cells in the current experiment, 2D autocorrelation maps were produced in the same manner as has been used to reveal the structure of medial entorhinal grid cell firing (Hafting et al. 2005). Autocorrelograms were constructed by taking the smoothed firing rate map and repeatedly correlating it with itself after it was shifted in successive 1-bin increments in the *X*- and *Y*-dimensions. Formally the autocorrelation procedure was defined as:

$$r(\tau_{x},\tau_{y})=\frac{\eta \sum \lambda (x,y)\lambda (x-\tau_{x},y-\tau_{y})-\sum \lambda (x,y)\sum \lambda (x-\tau_{x},y-\tau_{y})}{\sqrt{\eta \sum \lambda (x,y{)}^{2}-{\left(\sum \lambda (x,y)\right)}^{2}}\times \sqrt{\eta \sum \lambda (x-\tau_{x},y-\tau_{y}{)}^{2}-{\left(\sum \lambda (x-\tau_{x},y-\tau_{y})\right)}^{2}}}$$

Where *r*(*τ_x_*, *τ_y_*) is the autocorrelation between bins with an offset of *τ_x_* and *τ_y_*, *λ* (*x*, *y*) is the firing rate in the bin located at (*x*, *y*), and *η* is the number of bins over which the estimate was made.

As the repetition occurred along the *x*-dimension the central horizontal strip of the autocorrelogram was extracted and averaged to produce an overall 1D autocorrelogram for the ensemble of 15 cells.

#### Population Vector Analysis

The largest simultaneously recorded ensemble of CA1 place cells (*n* = 15) was characterized as a firing rate vector and used to estimate the rat's position during short periods of time (Wilson and McNaughton 1993; Fenton and Muller 1998; Fenton et al. 2008). A simple maximum correlation algorithm was employed to decode the animal's position as follows. A firing rate vector was constructed by arranging the firing rate maps into a 3D matrix with the 2 spatial dimensions represented on the *x*-*y-*axes and cell identity on the *z*-axis. A vertical line through the matrix yields the average firing rate vector across the ensemble for a particular location. These template vectors were then compared with the average of the current firing rate vector at each of the time steps, which ranged from 0.02 to 5 s. Errors reached a minimum at the 2-s integration window, and so this window was used for the statistical analyses. The decoded position of the animal was taken to be the location of the template firing rate vector that had the largest correlation with the current firing rate vector. If there was no cell activity during a given time step then no position was allocated for that time step. The reconstruction error was simply the absolute difference, for each of the 2 spatial dimensions, between the decoded and the actual position of the animal.

Collapsed firing rate maps were taken by dividing the environment into 4 equal parts, corresponding to the 4 compartments, and then combining homologous bins so that the result was a single, compartment-sized firing rate map in which each bin was the average of the 4 corresponding bins from each of the 4 compartments.

Scrambled firing rate maps were produced by again dividing the environment into 4 equal parts, and this time swapping homologous firing rate bins so that between-compartment differences were scrambled, while preserving spatial information from the within-compartment reference frame.

### Statistical Analysis

Statistical analyses were Pearson's *R*, Spearman's *ρ*, 1- or 2-tailed *t*-tests, 1- or 2-factor ANOVA and *z*-tests, all with a significance of *P* < 0.05.

### Histological Analysis

At the end of testing, the rats were deeply anesthetized with isoflurane and injected with sodium pentobarbital. They were then transcardially perfused with saline followed by paraformaldehyde (4%). The brains were removed and stored in paraformaldehyde (4%) for at least 1 week before sectioning. The brains were sectioned at 40 μm on a freezing microtome. The sections were then mounted and stained with Cresyl violet.

## Results

### Behavioral Analysis

Analyses of the paths taken by the rats during foraging were undertaken to look for evidence that the rats might have discriminated the compartments by spending more time in any of them (dwell time analysis) or by repeatedly entering any of them more or less often (re-entry analysis). Analyses were conducted on the first-ever exposure to the environment, and in relation to the context change.

#### First Exposure

In order to determine whether the rats showed an immediate preference for any of the compartments (e.g., those in the middle), which might indicate a behavioral ability to distinguish them, dwell times were computed for the 4 different compartments and converted into percentages of the total compartment time (which excluded time in the connecting corridor, which was not analyzed). Percentage times for compartments 1–4 (mean seconds ± S.E.M.) were, respectively, 27 ± 2, 23 ± 1, 22 ± 1, and 28 ± 1. However, because the 2 end compartments showed slightly higher dwell times than the 2 inner ones, these were combined and analyzed with a 1-tailed paired *t*-test. The mean percentage dwell time in the end compartments was 55% and in the inner compartments was 45%, which was significantly different [*t*_9_ = 2.27, *P* < 0.05]. There is thus a hint that the end compartments were slightly preferred (and by implication, discriminable from the middle ones).

We then looked at the number of re-entries into each of the 4 compartments, for the trial as a whole and also for each trial quarter, in case behavior changed during the course of the trial. The animals revisited each compartment numerous times (some more so than others) and there was no indication that any of the compartments were revisited more often by any of the animals. This was quantified by counting the entries in each quarter of the trial and comparing across compartments with a *χ*^2^ test: in none of the 40 analyses (10 rats × 4 trial-quarters) were there more entries into a given compartment than any of the others.

#### Context Change

We then analyzed behavior in response to the context-change manipulation, which was experienced by 5 rats. Initially a baseline trial was run with all the walls and floor of the multicompartment environment the same, followed by a context-change trial in which one of the middle compartments was changed to black, and then another baseline trial. Because either compartment 2 or 3 was used in different rats, the data were reflected where necessary so that the changeable compartment was always Compartment 3 in the analysis.

**Figure 2.**
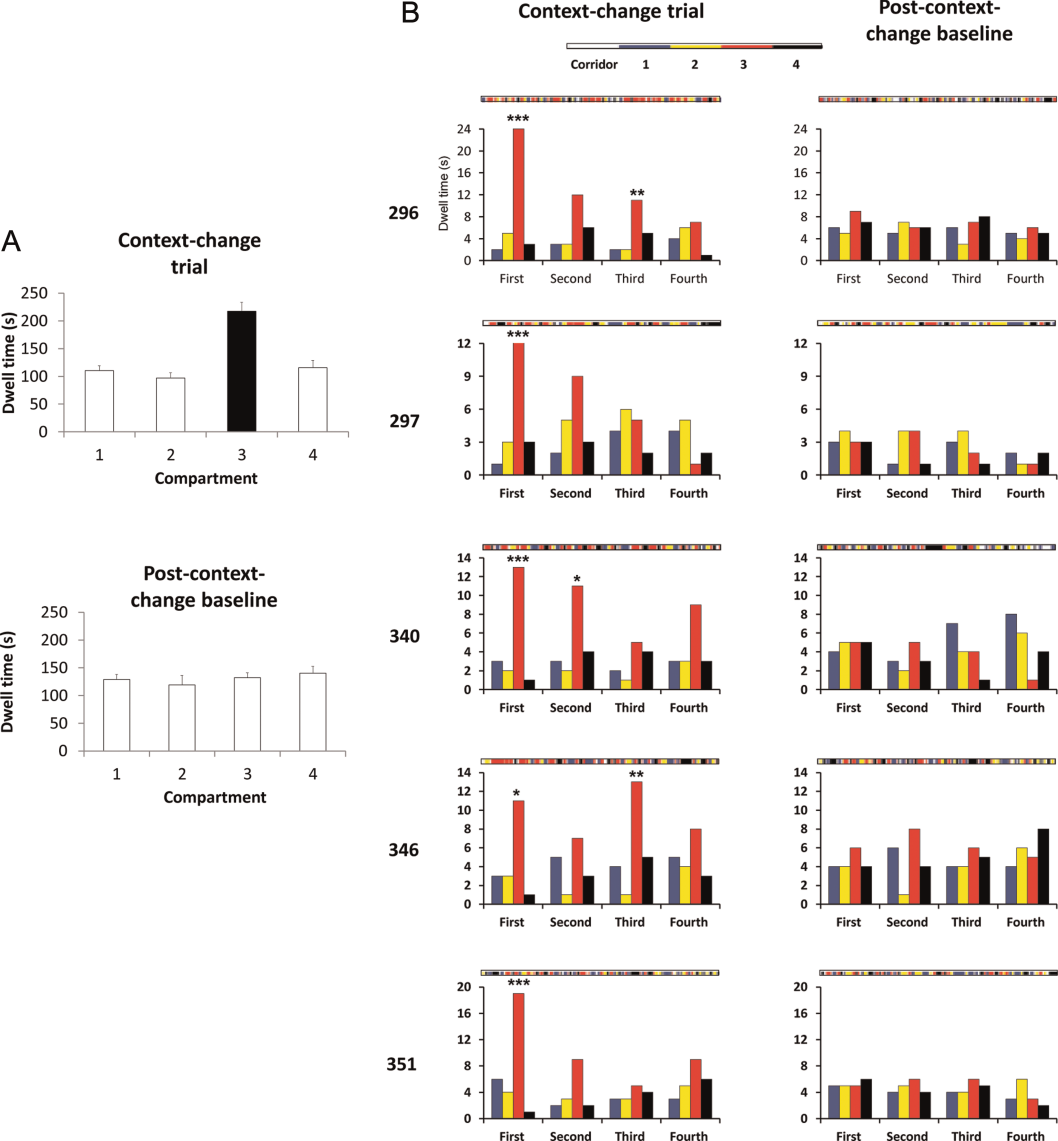
Analysis of dwell times and compartment re-entries in context-change trials and in the follow-up baselines. (*A*) Dwell time increased markedly in the compartment that was changed (black bar), whereas in the following trial, dwell time was not different in any of the compartments (including the had-been-changed one). (*B*) Compartment re-entries, broken down by trial quadrant. In the context-change trial (left column), re-entries increased markedly in the changed compartment (red bars), though this declined across the course of the session as the animals habituated to the change. In the follow-up baseline (right column), there was no difference in re-entries into any of the compartments, including the had-been-changed one.

The dwell times for the various conditions are shown in Table 1 and Figure 2. Change of the compartment from white to black caused a significant increase in dwell time, from a baseline average of 132 to 218 s (Fig. 2*A*). A 2-way ANOVA of compartment type against remapping condition showed a main effect of compartment type [*F*_3,48_ = 8.50, *P* < 0.001] and a significant interaction of trial type against compartment [*F*_6,48_ = 5.97, *P* < 0.001].

**Table 1:** Percentage dwell times in the 4 different compartments for rats in the pre- and postchange baseline conditions, or during the box-change trial when compartment 3 was changed from white to black (black border)

Compartment	1	2	3 (changeable)	4
Baseline 1	25 (3)	20 (1)	24 (1)	29 (3)
Context-change trial	21 (2)	18 (1)	42 (4)**	22 (2)
Baseline 2	24 (1)	22 (2)	25 (1)	27 (3)

Note: **P < 0.01.

We then looked at compartment re-entries, again broken down into trial-quarters, to see whether the rats entered any of the compartments more or less often during the context-change trial, or during the trial immediately afterward. A change occurring during the context-change trial that persisted into the following baseline trial would indicate 1) that the rats remembered the change, and 2) that they could tell the compartments apart. The results of this analysis are shown in Figure 2*B*. The change in compartment 3 was accompanied by a greatly increased number of visits to that compartment, reflected in the *χ*^2^ values which, for the 5 rats, respectively, were 50.58 (*P* < 0.0001), 14.69 (*P* < 0.001), 33.78 (*P* < 0.0001), 28.71 (*P* < 0.0001), and 28.10 (*P* < 0.0001). Visits to the changed compartment declined across the trial as the rats became habituated to the change. In the following trial, however, compartment 3 received no more or less visits than any of the other compartments, with *χ*^2^ values of 1.56, 1.90, 2.67, 2.77, and 0.7, none of which were significantly different from chance. Thus, the rats clearly noticed the change in compartment, but there was no evidence that they could remember and/or detect which had been the changed compartment on the follow-up trial.

### Single Neuron Analysis

Ten rats participated in the initial baseline test for repetition across environments. From these animals, 104 cells met our acceptance criteria and proceeded into the repetition analysis. Five of these rats were then tested in the context-change manipulation at intervals ranging from 2 to 28 days. From these animals, 29 cells entered the remapping analysis. Three of these rats, plus an additional newly implanted rat, entered into the wall-removal manipulation. From these animals, 39 cells were analyzed. Histological analysis confirmed that cells from 9 of the rats were recorded from hippocampal subfield CA1; the electrode track could not be identified in the remaining rat but the calculated depth putatively placed this in CA1 also.

We examined 3 questions: 1) To what extent do place fields show repeated patterns of place fields in each compartment, and does this change with increasing exposure? 2) Does a contextual (color/texture) change to one part of the environment result in purely local remapping or does the whole map change? And 3) Does a contextual (geometric) change to most of the environment cause remapping in one small part of it? As we show here, the evidence suggests that place cells form essentially identical representations of the different subcompartments, and encoding is predominantly local with weak rate-based discrimination.

#### Repetition of Fields in the Baseline Condition

**Figure 3.**
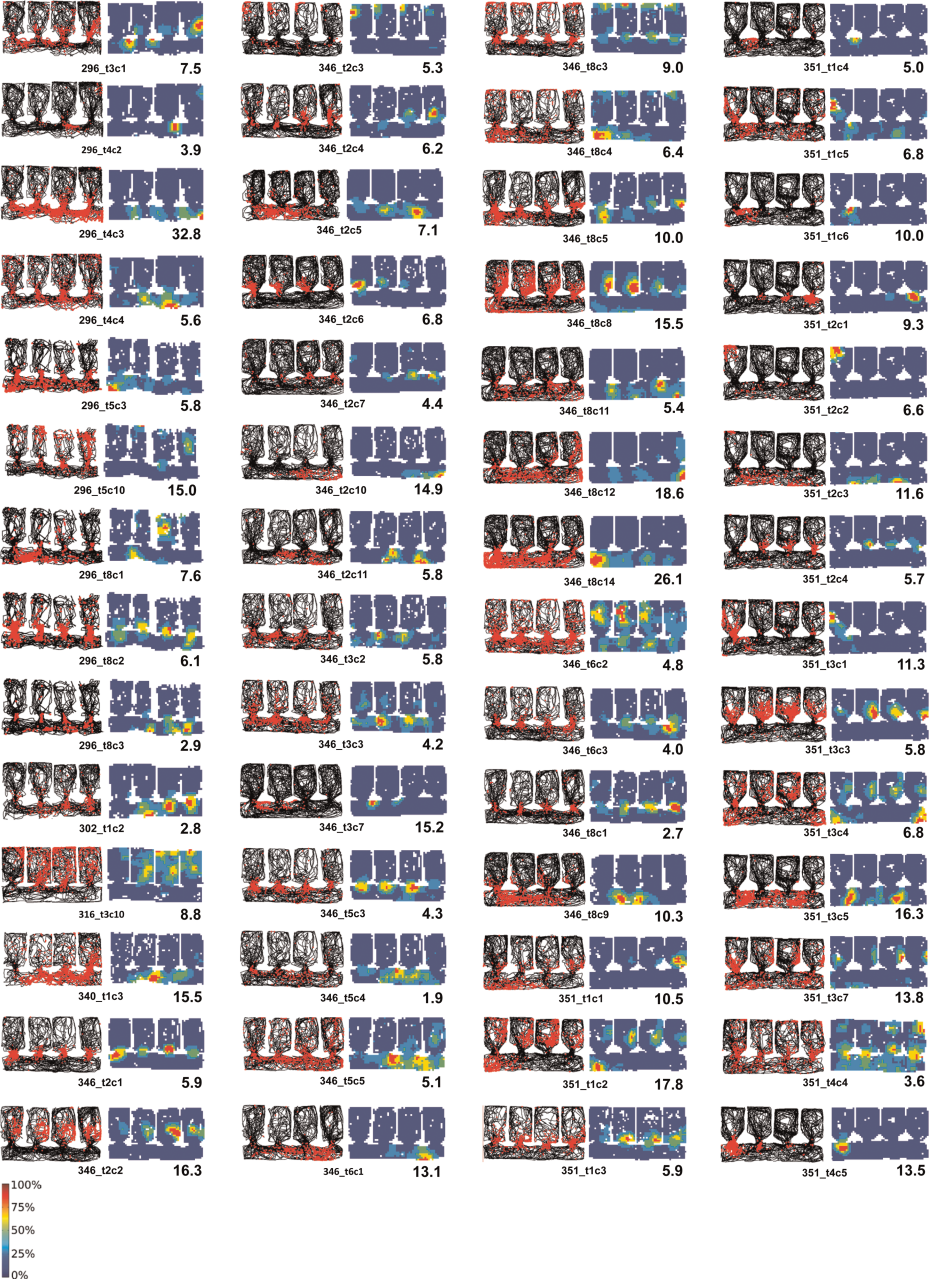
Montage of the firing maps of 56 place cells recorded in baseline conditions, shown as spike plots (top) or firing rate maps (bottom). For the spike plots, the path of the rat is shown in black, and the spikes shown as red dots. The condensation of spikes in a particular region of the environment comprises the place field of the cell. These cells were selected for showing activity in the corridor, as well as usually in the compartments. Note the prevalence of intense activity around the doorways. Even in the corridor, there was a high degree of repetition that followed the repetition of the environment. The firing rate maps show the same data expressed as a rate map, normalized for dwell time and for the peak rate of each cell. The peak rate in Hz is shown at the bottom right of each map and the key to the color plots is shown to the bottom left of the array.

**Figure 4.**
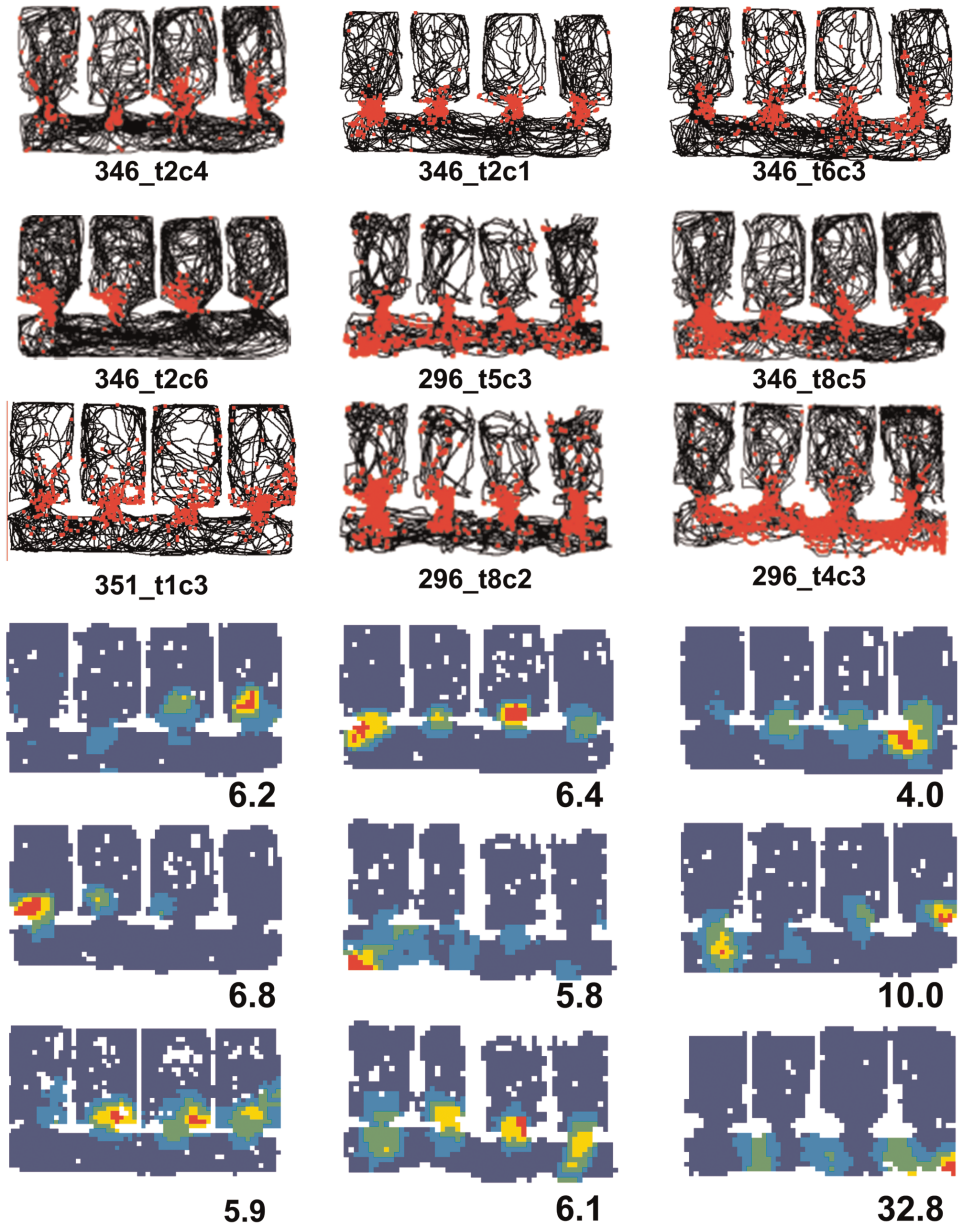
Intense activity around the doorways in 9 cells (plotted as for Fig. 3).

Place cell data (*n* = 104) from 10 rats were recorded from the baseline condition in the multicompartment environment, in which all compartments were identical (white). Examples of the overall firing patterns of place cells in the multicompartment environment are shown in the spike-plot montage in Figure 3. Place fields were nondirectional, as is generally the case in the open field. These cells are the subset that showed activity in the corridor. Activity of many cells was particularly intense around the doorways into each compartment (Fig. 4). Several cells showed unitary fields in the corridor, as would be expected given that the corridor was essentially a long rectangle. However, and interestingly, the firing in the corridors frequently showed a high degree of periodicity, mirroring the periodicity of the environment.

##### Place field location repetition analysis

**Figure 5.**
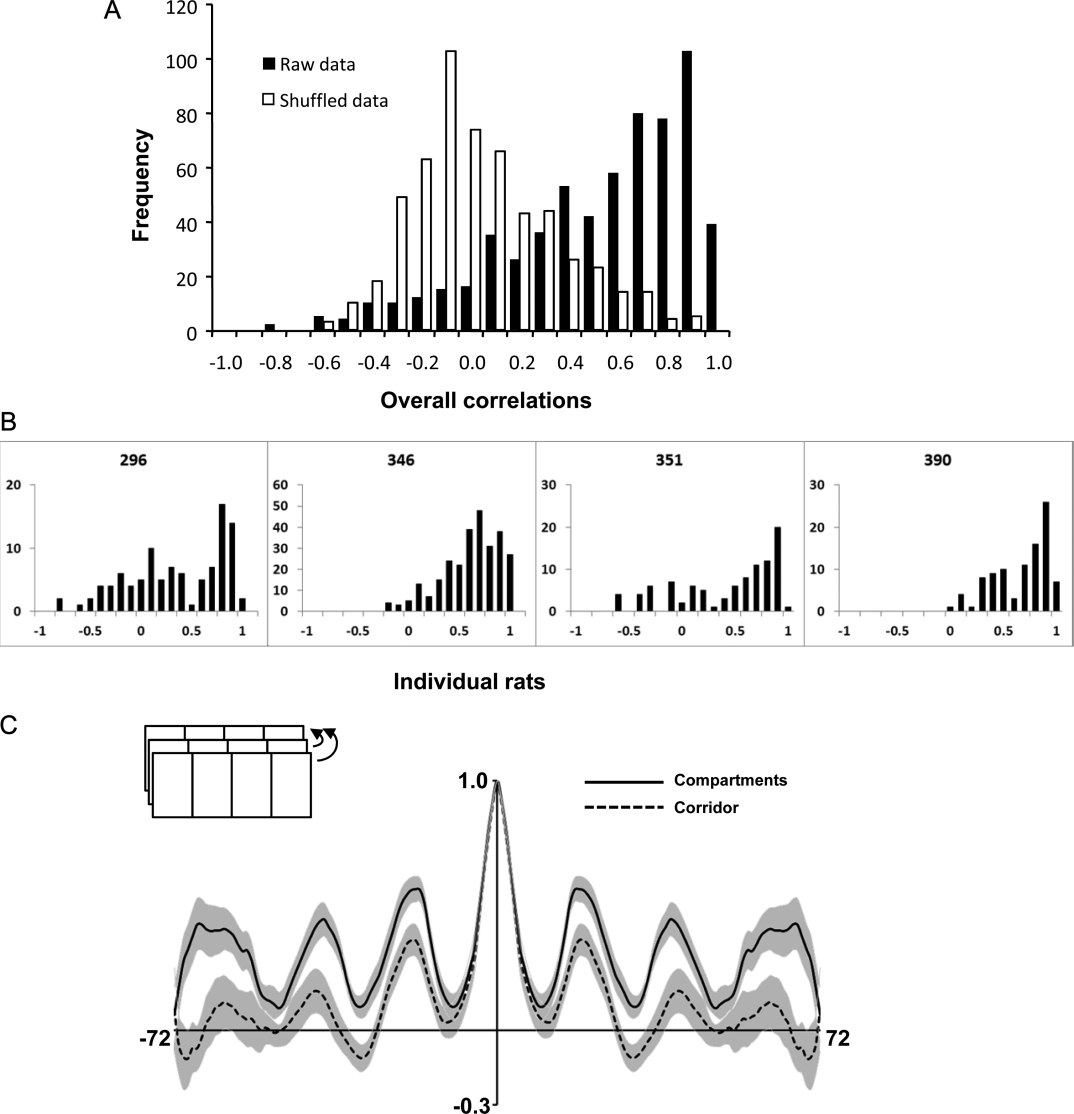
(*A*) Frequency histograms of the pairwise intercompartment correlations for the raw data (solid bars) and the shuffled data (hollow bars), showing a clear separation between the 2 distributions, reflective of the nonrandom relationship between place fields in different compartments. (*B*) The same correlation pattern held for individual animals, showing the generality of this effect. (*C*) The 1D autocorrelation plot, generated by progressively shifting the environment in the direction of the long axis (inset) and re-correlating at every step. The vertical axis represents the firing rate map correlation, with the central value at 1.0 (map correlated with itself). The horizontal axis indicates the extent of the environment in bins (72 in total). The shaded areas represent the standard errors. The correlations for the compartments (solid line) peaked at intervals corresponding to the width of a compartment, reflecting the underlying repetition of the place field map. This periodicity was also evident in the corridor fields (dotted line), although the peaks were slightly lower, reflecting the greater number of aperiodic place fields in the corridor.

Self-similarity was quantified in 2 ways. First, we performed bin-by-bin correlations between each pair of boxes, yielding a total of 6 correlation coefficient values per cell, and compared these correlations with those generated by randomly comparing unrelated firing fields. The fields used in the random analysis comprised the entire set of firing rate maps, pooled, from which samples were drawn without replacement. A frequency histogram of the shuffled data revealed correlations clustered just below zero, while the ordered data showed a range of correlations with a high number of high correlations (Fig. 5*A*), a pattern that was consistent across 4 example rats (Fig. 5*B*). This suggests that the fields in the different compartments were more similar than would be expected if they were forming independent representations.

These differences were quantified and statistically compared as follows (see Table 2 for the mean values). For the ordered data, the overall mean correlation coefficient across all 6 comparisons was 0.48 ± 0.03, a value consistent with previously obtained within-field between-identical session correlation values (Anderson and Jeffery 2003). For the shuffled data, the overall mean correlation was 0.01 ± 0.01. A 1-tailed *t*-test comparing these values (averaged across compartment-pairs for each cell) found a highly significant difference [*t*_206_ = 15.53, *P* < 0.0001]. This degree of across-compartment correlation in the ordered dataset stands in contrast to the report by Skaggs and McNaughton (1998) that about 50% of cells “remapped” between 2 identical compartments. While there was some evidence of bimodal distributions in the frequency histograms of correlations (Fig. 5*A*), in general, most cells showed a high degree of repetition across the compartments.

**Table 2:** Firing rate map correlations between each of the 4 compartments and each of the others, both for the ordered data and for the shuffled data

	Ordered data			
Compartment	1	2	3	4
1	–	0.46	0.43	0.37
2		–	0.60	0.46
3			–	0.53
4				–
	Shuffled data			
Compartment	1	2	3	4
1	–	−0.04	0.01	0.05
2		–	0.01	0.01
3			–	0.00
4				–

Note: The correlations for the shuffled data were low, as would be expected, whereas for the ordered data they were high, indicating a high degree of self-similarity in the firing fields.

We then looked at whether the correlations in the ordered data were varied by compartment-pair: a 1-way ANOVA was significant [*F*_5,618_ = 4.8, *P* < 0.001]. A post hoc test (Tukey's) found that the correlation between compartments 2 and 3 was, at 0.6, significantly higher than for any of the other pairs except 3 versus 4 (*t*'s all >2.88, *P* < 0.01). Thus, there was a tendency for firing in the 2 middle compartments to be slightly more similar than the other pairs.

The second repetition analysis involved taking the firing rate maps and performing a 1D spatial autocorrelation, which entailed progressively shifting each map in bin-by-bin steps and re-correlating with the original at each step to produce an autocorrelation plot (Fig. 5*C*). This was done separately for the compartment zones and the corridor zones. The resulting compartment plot was periodic, as expected from the repeating fields: this was also true for the corridor, although the overall degree of correlation for the corridor was significantly lower [*t*_284_ = 9.05, *P* < 0.0001]. Thus, there was a high degree of periodicity in the firing patterns of the cells, reflecting the self-similarity of the compartments.

##### Rate remapping analysis

Although the spatial patterns of firing were similar across compartments, place cells are sometimes known to exhibit a phenomenon known as “rate remapping” in which the firing rate rises or falls in response to a change in environment (Hayman et al. 2003; Leutgeb et al. 2005). We saw several examples of what looked like possible rate remapping (Fig. 4) and so undertook a statistical analysis of whether there might be something like a rate code for compartment identity. We did this in 3 ways: first, by using a neighborhood analysis to look for progressive rate changes across the compartments; second, by looking at an ensemble of simultaneously recorded cells to see whether the pattern of firing rates carried any information about compartment identity; and third by examining the stability of rank-ordered compartment peak-rates between baseline sessions.

**Figure 6.**
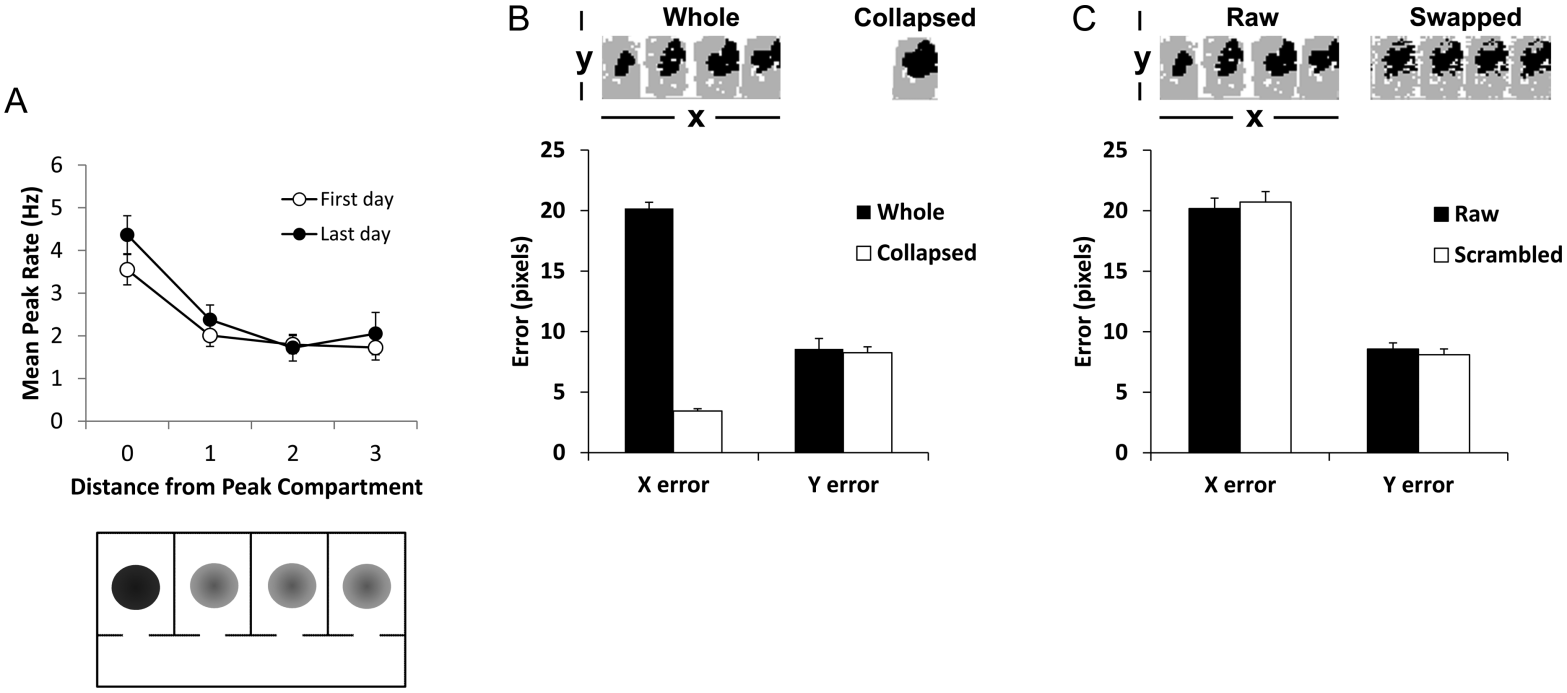
Rate coding analysis, to see whether firing rates might contain information that could be used to disambiguate compartments. (*A*) Change in peak firing rate of place cells as a function of distance from the compartment having the peak rate, for naive rats (“First day”; open circles) or rats with extensive experience of the environment (“Last day”; closed circles). Below is shown a plan view of the apparatus, filled circles represent place fields from a single hypothetical place cell, illustrating the result shown above for comparison to the predictions in Figure 1. (*B*) Reconstruction error analysis, based on an ensemble of 15 simultaneously recorded cells, in which the firing rate population vector was used to reconstruct the position of the rat, and this position compared against the rat's actual position in the *X*- and *Y*-dimensions. The data for the whole 4-compartment environment were compared against the same dataset collapsed in the *X*-dimension (see inset) onto a single, composite compartment (thus losing compartment-specific information). For the whole-environment dataset, the *X*-error was much great than the *Y*-error, which reflects the ambiguity in the firing rate information about which environment the rat was in. When the data were collapsed onto a single-compartment reference frame, the *X*-error was slightly less, probably because the *X*-dimension was slightly but significantly less than the *Y*-dimension and there was thus less room for error. (*C*) The raw data were compared against the same data in which homologous bins in the 4 compartments were exchanged, so that compartment-specific rate information would be dispersed while spatial information common to all 4 compartments would be preserved. Although in both datasets the errors in the *X*-dimension were much larger than those in the *Y*-dimension, this error was even greater for the scrambled data than the raw data, suggesting a slight degree of compartment-specific information contained in the firing rate maps.

In the first—neighborhood—analysis, we assigned neighborhood relations to the compartments as follows: any compartments adjacent to the peak were labeled “1,” any that were next-but-one were labeled “2” and any that were 2 compartments away (for the subset of cells with peak fields in an end compartment) were labeled “3.” For cells in which there were 2 compartments occupying the “1” position, these values were averaged. We then analyzed firing rates as a function of this ordinal distance from the peak-rate compartment for both first day and last day of recording (see Fig. 6*A*). A 2-factor (day and compartment distance) repeated-measures ANOVA revealed a main effect of compartment distance [*F*_2,64_ = 15.1, *P* < 0.001, but no main effect of day or significant interaction. Unsurprisingly, for both first and last day, post hoc *t*-tests revealed significant differences in rate when the peak compartment was compared with compartments at a distance of 1 (*P* < 0.05, Cohen's *d* for first day = 0.64, Cohen's *d* for last day = 0.55), 2 (*P* < 0.05, Cohen's *d* for first day = 0.77, Cohen's *d* for last day = 0.76), or 3 compartments from the peak (*P* < 0.05, Cohen's *d* for first day = 0.79, Cohen's *d* for last day = 0.63). However, no other post hoc tests were significant (maximum Cohen's *d* = 0.22, 1 vs. 2 on last day). Thus, our neighborhood analysis provided no evidence of any modulation of peak rates by distance beyond the peak compartment.

In our second analysis, we undertook an ensemble analysis of rate coding. Although there was not a large systematic change across the compartments, it is possible that there are subtle consistencies in compartment-specific firing rate distributions that could amount to a rate code for compartment identity, even though this was not obvious by eye. We investigated this by taking the biggest simultaneously recorded ensemble of cells in our dataset (*n* = 15) and using information about the firing to reconstruct the rat's position, using the population vector analysis described in the Materials and Methods section. In brief, this process works by initially determining the unique firing rate pattern of the ensemble for each bin in the environment (the vector of firing rates) and then using it to predict the position of the rat based on the momentary firing rate pattern in the ensemble at each time step. If compartment-specific information is contained within the firing rates, then it should matter whether the compartments are in their usual order or whether the bins in the firing rate map are swapped between compartments.

Reconstructed position was compared against actual position separately for the *X*-dimension, which is the long axis of the environment (parallel to the corridor), and the *Y*-dimension. The errors were compared between the raw data and the same data in which compartment identity was rendered irrelevant, either by collapsing the 4 compartments into 1 (Fig. 6*B*) or by swapping homologous bins between compartments so as to lose compartment-identity information while retaining intracompartment positional information (Fig. 6*C*). An ANOVA revealed main effects of dimension [*F*_1,3845_ = 56.0, *P* < 0.0001] and of collapsing the compartments [*F*_1,20910_ = 305, *P* < 0.0001], but no main effect of swapping the pixel identities. For the raw data alone, errors in the *X*-dimension were on average 20.18 ± 0.86 bins, considerably greater than in the *Y*-dimension at 8.57 ± 0.51 bins [*t*_225_ = 11.30 *P* < 0.0001], as would be expected given the high degree of field repetition across compartments and the consequent ambiguity about where the rat was. However, when the compartments were collapsed into one, *X*-errors actually became slightly smaller (3.44 ± 0.18) than *Y*-errors (8.26 ± 0.18 bins), a difference that was highly significant [*t*_225_ = 15.73 *P* < 0.0001] and likely reflects the smaller size of the compartment in the *X*-dimension (30 cm) than the *Y*-dimension (40 cm).

The bin-swapping procedure had the effect of rendering compartment-specific information irrelevant, and was undertaken to see whether there was any such information present, implicitly, in the raw data. In the swapped dataset, *X*-errors exceeded *Y*-errors by a considerable amount (19.84 ± 0.06 bins for *X* and 5.00 ± 0.25 bins for *Y*) that was significant [*t*_736_ = 22.65 *P* < 0.0001]. There was no significant difference between *X*-errors in the swapped dataset and *X*-errors in the raw dataset. The conclusion from the rate analyses is that there is a slight amount of rate coding in the firing rate maps, and a hint of a tendency for this pattern to increase with experience. However, the effects are mild in degree compared with previous studies (e.g., Hayman et al. 2003), and it does not seem that firing rate is a substantial marker of global spatial location, though it may provide a modulating influence.

In our third rate analysis, for each cell having 2 baseline recordings (*n* = 68), we examined whether the pattern of peak rates across the 4 compartments in the first baseline trial would match the pattern in the subsequent baseline trial. This analysis thus tests stability of the peak rates across sessions, indicating a possible stable rate code for compartment identity. Firing across the 4 compartments was ranked such that the compartment with the peak firing was coded as 1, the next highest as 2, and so on. Of the 68 cells recorded in the repeated-baseline conditions, 13 had the same peak rate pattern in the 2 baseline sessions. A binomial test confirmed that 13 matches was significantly higher (*P* < 0.001) than the 3 matches predicted by chance (4 compartments provide 24 unique combinations of firing rate order). Thus, the data demonstrate a degree of stability in terms of the order of peak firing in the compartments between baseline trials. In sum, our 3 analyses of peak rate reveal a weak modulation of peak rate within a session and a degree of stability of the pattern of compartment peak rates across baseline sessions.

#### Context-Change Trials

After the rats had had considerable experience of exploring the multicompartment environment across several days of recording, a remapping test was carried out involving 29 cells from 5 rats in 5 context sessions (respectively, *n* = 5,*n* = 1,*n* = 2, *n* = 15, *n* = 6). In this test, 1 of the 2 middle compartments was changed from white to black (Fig. 1*B*). There were 2 questions of interest: 1) Did firing patterns change in the altered compartment, and 2) Did firing change in the regions of the environment outside the altered compartment. In other words, were there any nonlocal changes in the firing of the cells?

**Figure 7.**
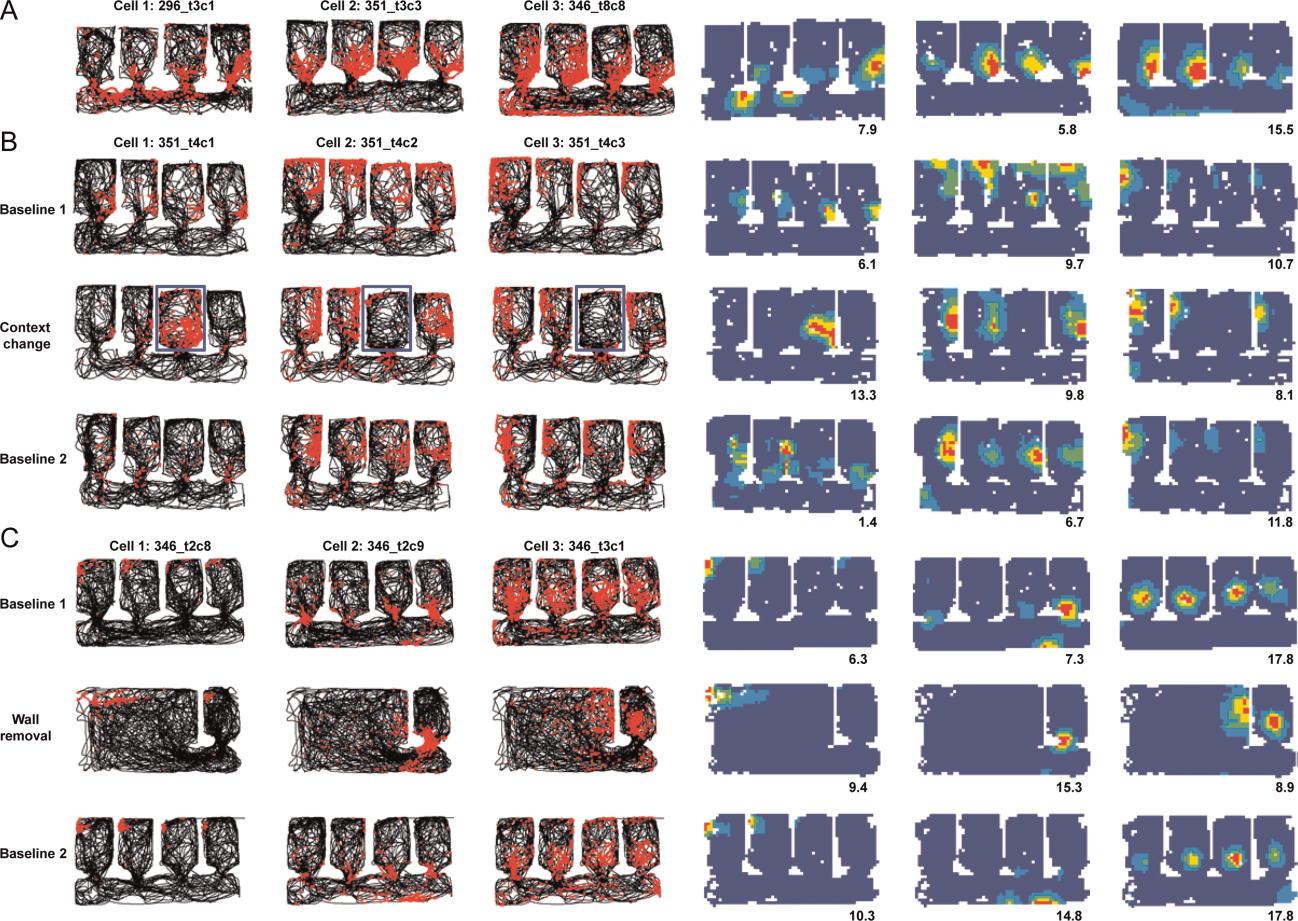
Remapping in place cells, showing red spikes superimposed on the black path of the rat, as in Figure 3. (*A*) Rate remapping, in which some cells appeared to differentiate the compartments by varying their firing rate. (*B*) The response of 3 cells to the context-change manipulation. The changed compartment is outlined in blue. Note that the change in firing was local to the changed compartment and did not spread beyond its confines. (*C*) The response of 3 cells to the wall-removal manipulation. Again, the change was local, affecting only the parts of the environment that were changed (in this case, all but the right-hand compartment).

Figure 7*B* shows the effects of this manipulation on 3 cells, in which it can be seen that the place cell firing pattern in the altered compartment was different, indicating remapping. By contrast, the firing in the remaining compartments was unaltered.

Remapping was quantified by 1) Comparing spatial bin-by-bin correlations, and 2) Comparing the peak firing rates in the compartments across the different trial types.

##### Spatial correlation analysis

**Figure 8.**
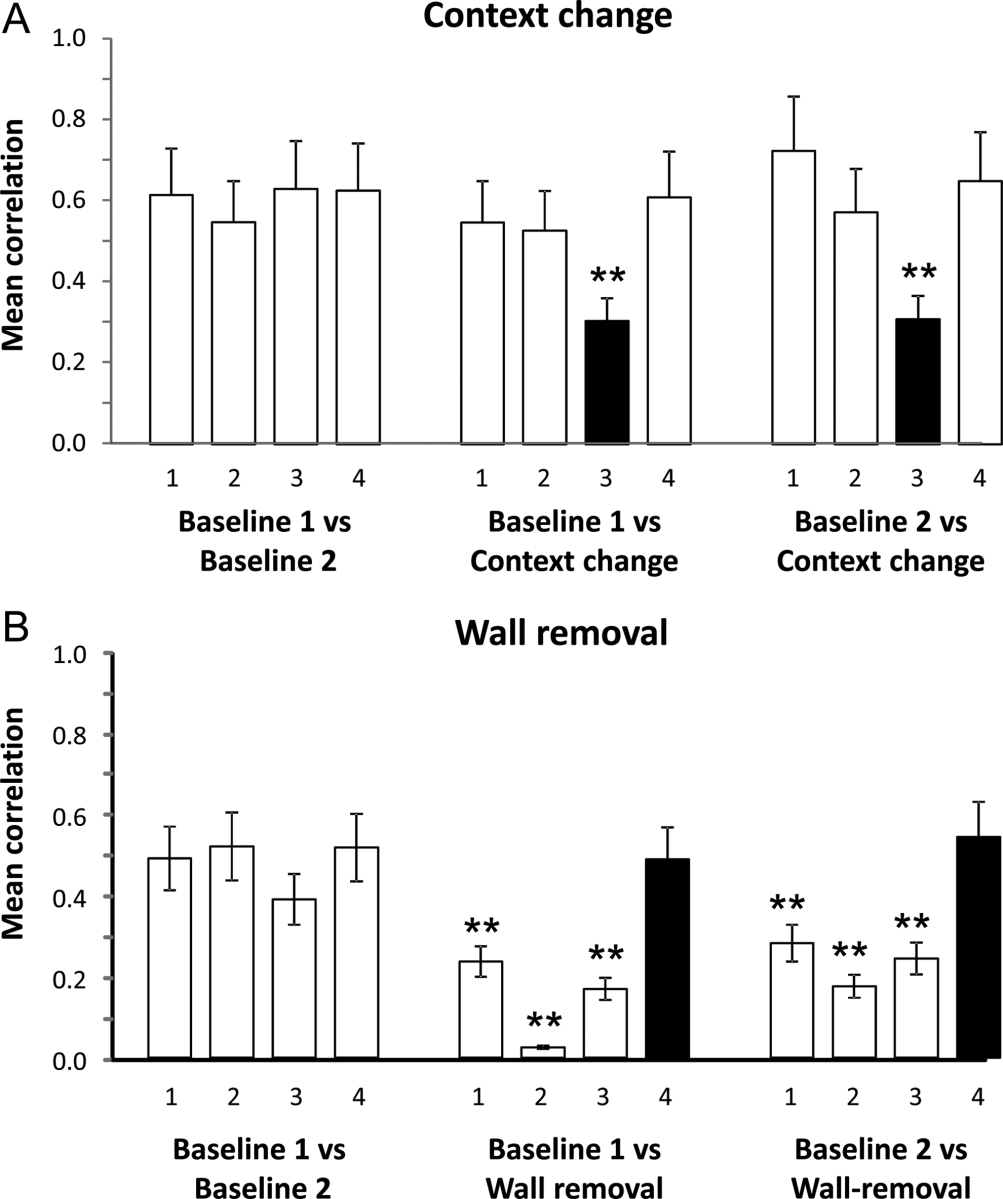
Within-compartment spatial bin-by-bin correlations between the first and last baseline conditions, or between each baseline and the environment-change trials. (*A*) Context-change manipulation. (*B*) Wall-removal manipulation. The compartments of interest are labeled with black bars. In the context-change trials, this is compartment 3, which was changed from white to black: correlations with this compartment dropped markedly in the context-change manipulation. In the wall-removal trials, this was compartment 4, which was the only compartment not to be changed, and likewise the only compartment in which correlations did not drop significantly. **P < 0.001.

The results of the bin-by-bin spatial correlations, in which firing in each compartment in each condition was compared with firing in the same compartment in the other conditions, are shown graphically in Figure 8*A* and numerically in Table 3. The correlations in the changed compartment showed a large decrease, as expected, reflecting the reorganization of firing that took place in these compartments. Notably, however, the correlations in the other compartments did not change. This was quantified with a 2-way ANOVA comparing compartment against trial type, which showed no effect of trial type [*F*_2,6_ = 2.4, NS] but a significant effect of compartment [*F*_3,6_ = 8.7, *P* < 0.001] and a significant interaction [*F*_6,312_ = 2.9, *P* < 0.01]. Post hoc pairwise comparisons revealed that all correlations involving the context-change compartment on the context-change trial were significantly different (all *t*-values >2.29, *P*-values <0.05) except for the 2 correlations between the 2 baseline trials and the remapping trial, which were (as expected) both low and did not differ. By contrast, all other correlations were not significant. Therefore, changing the sensory qualities of a compartment significantly altered firing in that compartment, but only on that trial and only in that compartment. There was thus no nonlocal effect of the context change.

**Table 3:** Statistical analysis of the trial-pair correlations

	Color change spatial correlations (mean±S.E.M)			
Compartment	1	2	3	4
Baseline 1 vs. Baseline 2	0.60 (0.07)	0.56 (0.06)	0.63 (0.05)	0.63 (0.04)
Baseline 1 vs. color change	0.57 (0.08)	0.56 (0.07)	0.28 (0.08)**	0.61 (0.06)
Baseline 2 vs. color change	0.72 (0.04)	0.59 (0.08)	0.28 (0.07)**	0.66 (0.06)
** *P* < 0.001				
	Wall removal spatial correlations (mean ± S.E.M)			
Compartment	1	2	3	4
Baseline 1 vs. Baseline 2	0.49 (0.08)	0.52 (0.08)	0.39 (0.06)	0.52 (0.08)
Baseline 1 vs. wall-removal	0.24 (0.04)**	0.03 (0.00)**	0.17 (0.03)**	**0.49 (0.08)**
Baseline 2 vs. wall-removal	0.28 (0.05)**	0.18 (0.03)**	0.24 (0.04)**	**0.54 (0.09)**

Note: BS1, baseline 1; BS2, baseline 2; CC, context change; WR, wall removal. For the context-change trials, only correlations involving the changed compartment in the context-change trials (**black border**) were significantly different from the baseline comparisons. For the wall-removal trials, the changed compartments (1–3) all showed a decrease except for the BS1-BS2/BS2-WR comparison, which was not different. Correlations involving the unchanged compartment (**black borders**) did not differ from baseline. *P< 0.05, **P < 0.01.

##### Peak rate analysis

**Figure 9.**
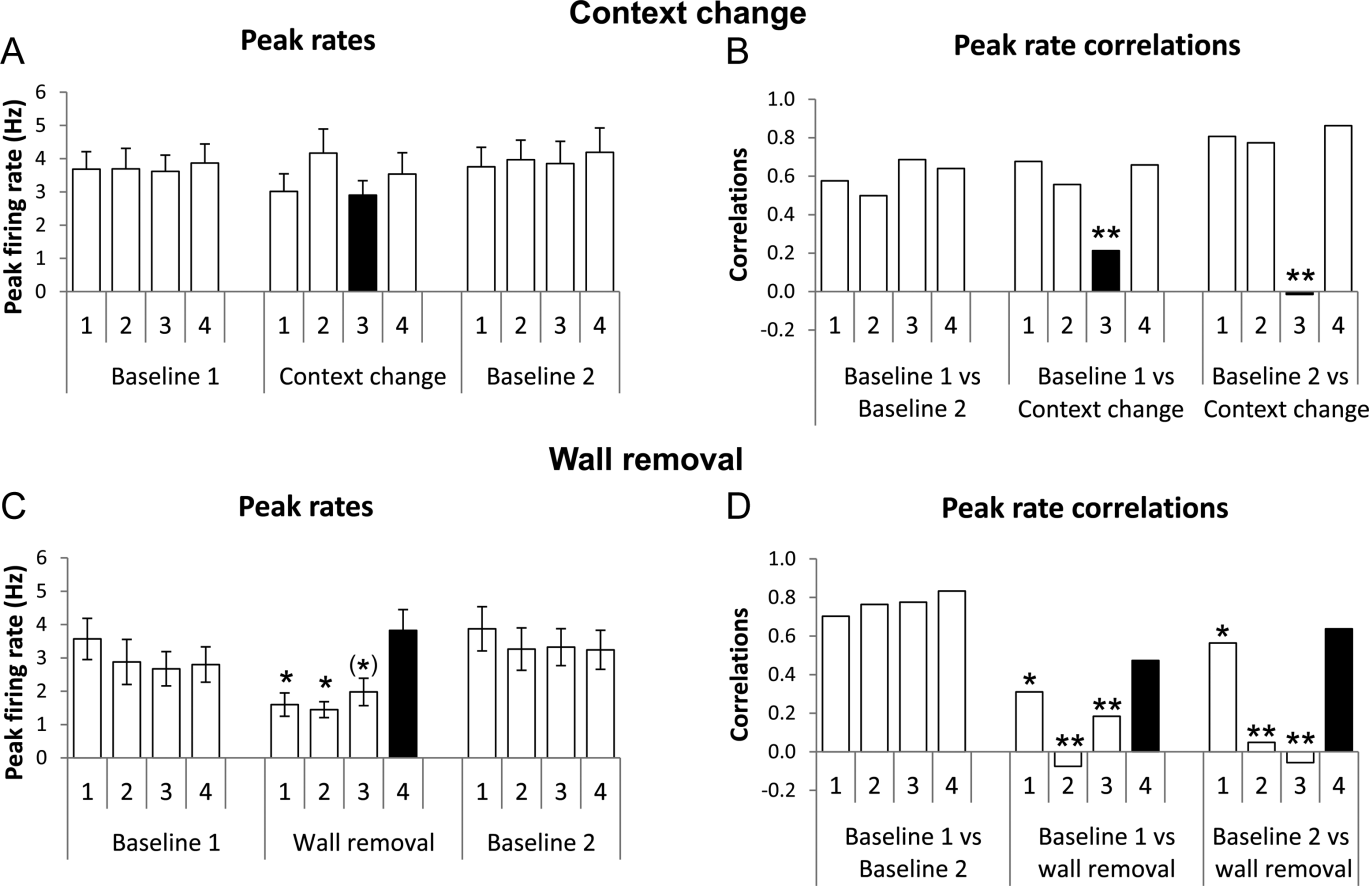
Changes in peak rates of place cell firing when the environments were changed. (*A*) and (*B*) are from the context-change manipulation, (*C*) and (*D*) from the wall-removal one. (*A*) In the context-change trials, overall peak rates became slightly more variable between the baseline conditions and the context-change condition, but this was not significant, even for the compartment that was actually changed (black bar). (*B*) Within-cell between-condition correlation of peak rates, however, showed a significant change, indicating variability of firing rate in the changed compartment (black bar) relative to the unchanged compartments/conditions. (*C*) Removal of the walls separating compartments 1–3 caused a drop in the overall firing rates in those regions of the environment (black bars), but not in the compartment that remained enclosed. (*D*) Correlations in peak rate firing dropped in the 2 central changed compartments in the changed condition when compared with the unchanged condition—the end compartment retained a relatively high correlation, perhaps because 3 of its 4 walls still remained. The firing rates in the unchanged compartment remained highly correlated throughout. *P < 0.05, (*)*P* < 0.05 only for the second comparison (wall-removal vs. second baseline).

Remapping was also explored by looking at how firing rates might have changed in response to the manipulation. The results of the peak rate analysis are shown in Figure 9*A*. Overall firing rates did not change significantly between the baseline and context-change conditions, even for the compartment in which the change was actually made, as evidenced by a 2-way repeated-measures ANOVA of compartment against trial type revealing no effect of compartment [*F*_3,83_ = 0.49, NS], no effect of trial type [*F*_2,84_ = 0.33, NS], and no interaction [*F*_6,168_ = 0.87, NS].

It may be, however, that peak rates changed either upward or downward on a cell-by-cell basis, which would not have been evidenced by a global peak rate analysis. Therefore, we correlated firing rates on a cell-by-cell basis between each trial pair, generating a global peak rate correlation across the 29 cells (Table 4). This resulted in a notable drop in rate correlation for compartment 3 in the context-change condition, when compared with the other conditions. This was quantified by calculating lower-bound confidence intervals (CI) for the aggregated data from the unchanged compartments only. The correlation values involving the compartment that changed lay outside the 99% CI, while all other values (including that compartment in the baseline comparisons) lay within the 95% limits. Therefore, peak rates were more altered for the changed compartment than for the others, and the effect was local.

**Table 4:** Correlations between the peak firing rates for individual cells when compared across the various trial conditions

Context-change peak-rate correlations				
Unchanged compartments: mean = 0.67; stdev = 0.11; 95% CI = 0.44; 99% CI = 0.33				
Compartment	1	2	3	4
Baseline 1 vs. Baseline 2	0.58	0.50	0.69	0.64
Baseline 1 vs. context change	0.68	0.56	**0.21**^b^	0.66
Baseline 2 vs. context change	0.81	0.77	**−0.02**^b^	0.86

Wall removal peak-rate correlations				
Unchanged compartments: mean = 0.70; stdev = 0.13; 95% CI = 0.44; 99% CI = 0.31				
Compartment	1	2	3	4
Baseline 1 vs. Baseline 2	0.70	0.76	0.78	0.83
Baseline 1 vs. wall removal	0.31^a^	−0.08^b^	0.18^b^	**0.47**
Baseline 2 vs. wall removal	0.56	0.05^b^	−0.06^b^	**0.64**

Note: Confidence intervals (CI) were calculated by aggregating the values for the unchanged compartments. For the context-change trials, only correlations involving the changed compartment in the context-change trials (**black border**) lay below the lower CI. For the wall-removal trials, the middle 2 compartments showed a large drop in correlation, compartment 1 (which retained 3 of its original walls) only showed a slight drop when Baseline 1 was compared with the wall-removal trial, and the unchanged compartment (**black borders**) showed no significant change.

^a^Below 95% CI.

^b^Below 99% CI.

#### Geometry-Change by Wall Removal Trials

The locality of the remapping effect in the context-change trials may have been due to the small area of the changed compartment relative to the whole environment. Accordingly, we reversed the conditions and made a change to the whole of the environment except one of the compartments. The reasoning was that if place cells show nonlocal encoding, then the global change ought to induce local encoding changes, even for the part of the environment that remained unaltered. The global change was made by removing those internal walls that had created compartments 1–3 (Fig. 1*C*). The effects of the wall removal on the firing of 3 place cells is shown in Figure 7*C* where it is evident that firing in the unchanged compartment is essentially unchanged between the baselines and the wall removal trial, and the firing in the merged space breaks down.

##### Spatial correlation analysis

The results of the bin-by-bin spatial correlations are shown in Figure 8*B*. The correlations in the merged space previously occupied by compartments 1–3 showed a large decrease, as expected, reflecting the reorganization of firing patterns that had taken place. The correlations in the unchanged compartment (compartment 4) did not change. This was quantified with a 2-way ANOVA comparing compartment against trial type (removing empty cells due to no firing), which showed a main effect of trial type [*F*_2,54_ = 18.5, *P* < 0.0001], a main effect of compartment [*F*_3,81_ = 5.60, *P* < 0.01], and a significant interaction [*F*_6,162_ = 4.11, *P* < 0.001]. Post hoc paired comparisons revealed that compartments 1–3 showed a significant decline in correlation between both baselines and the wall-removal trial (all *t*-values >2.08, *P*-values <0.05) whereas compartment 4, the one that remained enclosed, did not.

##### Peak rate analysis

The results of the peak rate analysis are shown in Figure 9*C*,*D*. A 2-way repeated-measures ANOVA of compartment against trial type revealed no effect of compartment [*F*_3,6_ = 1.16, NS] but a main effect of trial type [*F*_2,6_ = 3.86, *P* < 0.05] and a significant interaction [*F*_6,228_ = 6.67, *P* < 0.001]. Post hoc paired comparisons for virtual subcompartments within the merged space showed that compartment 1 firing rates decreased significantly between the first baseline and the wall-removal trial, dropping from 3.57 ± 0.47 Hz to 1.60 ± 0.43 Hz (*t*_38_ = 3.24, *P* < 0.01) and rising again between the wall-removal trial and the final baseline, to 3.87 ± 0.51 Hz (*t*_38_ = −4.15, *P* < 0.001); “compartment 2” decreased between baseline 1 and wall removal from 2.88 ± 0.45 Hz to 1.45 ± 0.41 Hz (*t*_38_ = 1.95, *P* < 0.05) and rising again to 3.26 ± 0.37 in the second baseline (*t*_38_ = −2.71, *P* < 0.01), and “compartment 3” showed an not-quite-significant decrease between the first baseline and the wall removal trial, from 2.67 ± 0.30 to 1.98 ± 0.48 Hz (*t*_38_ = 1.17, NS), but a significant increase again in the final baseline to 3.32 ± 0.03 Hz (*t*_38_ = −1.90, *P* < 0.05). By contrast, the firing rates in compartment 4, which remained unaffected by the wall-removal manipulation, did not change, showing in fact a slight but insignificant increase in rate from 2.80 ± 0.39 Hz in the first baseline to 3.83 ± 0.55 Hz in the wall-removal trial (*t*_38_ = −1.71, NS), and a slight but insignificant decrease again to the final baseline of 3.24 ± 0.41 Hz (*t*_38_ = 1.13, NS). Thus, the wall-removal manipulation caused a generalized decrease in firing rates in the merged compartments, but this change did not affect firing rates in the unchanged compartment.

We also, as with the context-change manipulation, looked at peak rate behavior on a cell-by-cell basis, by generating peak rate correlation values for the whole population of cells (Fig. 9 *C,D*), and comparing these across trial types (Table 4). CIs were calculated as for the context-change trials, using only the unchanged compartment data (from all 4 compartments in the baseline conditions, and from compartment 4 in the wall-removal condition) and the changed-compartment values were compared against these. The 2 middle compartments showed a large drop in firing rate correlation, to below the 99% CI in both comparisons. Compartment 1 showed a slight drop in only one of the comparisons, perhaps because more of its original walls still remained. Compartment 4, the unchanged compartment, did not show a significant drop in firing rate correlation. Thus, the large changes that affected the changed compartments did not propagate to the unchanged compartment.

## Discussion

This study examined whether hippocampal place cells would be able to use path integration to disambiguate identical subregions of a multicompartment environment, devoid of extramaze cues, in which rats could freely forage. We recorded place cells in a curtain-enclosed environment comprising 4 adjacent identical compartments separated from a common connecting corridor by doorways. We found that there was little difference between the spatial location of place fields in the compartments, even for very experienced rats. A change to one of the compartments did not affect firing in the unchanged regions, and a change to all the other compartments except one did not induce a change in that one unchanged compartment. Thus, although the rats were able to walk freely between all the compartments, and so amass path integration information about the different compartments and their spatial relations to each other, this did not cause differential location of place fields in the different compartments. There was, however, a slight degree of firing rate variation that had the potential to slightly disambiguate compartments. These results thus reveal the limitation of the path integration system to, under self-driven foraging conditions, allow place cells to develop distinct representations of different, but visually similar, bounded regions of space. Below, we discuss how the inputs to place cells might cooperate to produce such a pattern.

This finding of place field repetition stands in apparent contradiction to some previous reports that place cells recorded during foraging could show such disambiguation (Skaggs and McNaughton 1998; Tanila 1999; though see Fuhs et al. 2005). The 2 studies reporting discrimination (Skaggs and McNaughton 1998; Tanila 1999) found that about 50% of place cells remapped—that is, switched on or off their fields, or altered their fields—as a rat moved between 2 identical compartments. There were several methodological differences that may be relevant to the different findings—e.g., the compartments in the Skaggs and McNaughton (1998) study were a little larger than ours (58 × 61 cm vs. 30 × 40 cm) and they were lit by a (potentially orienting) point source whereas our environment was diffusely lit. However, because the compartment size differences are small (compared with studies revealing differences in place field responses, (Fenton et al. 2008), and that light source differences both produce similar visual environments, it is more likely other factors drive the repetition of fields. In our study, to minimize the potential use of local cues, wall sections were interchanged, the floor rotated and wiped, and the apparatus moved by one compartment width within the room between every trial. A similar procedure was used by Skaggs and McNaughton (1998) of changing the floor and switching the boxes positions in the recording room. Skaggs and McNaughton (1998) did not find that place cells followed the box, rather they followed the position of the box (North vs. South), indicating that local cues from the box cannot directly account for the separate coding of the 2 compartments in Skaggs and McNaughton (1998). Arguably the most probable cause for the discrepancy between our data and other studies is the number of compartments used (2 vs. 4). Environments with only 2 compartments would be easier to learn because the place cell network only needs to organize 2 states, triggered by cues that are unique to only one of the compartments. Once in the compartment the rat must maintain the separation between place cell ensemble firing patterns for each compartment. With 2 compartments there are only 2 states, one to suppress, the other to maintain. With more than 2 compartments there are more ensemble states to suppress.

It has been proposed the hippocampus uses sensory information from both external sources (e.g., vision) and internal movement related sources (e.g., otoliths) as inputs to drive population activity in an attractor network of place cells (Samsonovitch and McNaughton 1997; McNaughton et al. 2006). The attractor network dynamics are thought to help ensure the stability of the place cell ensemble re-activation of firing patterns in the face of complex or subtle changing sensory information (see Nakazawa et al. 2002; Jezek et al. 2011). In discriminable environments, the place representation patterns are separated via sparse coding in the dentate (McNaughton et al. 2006). Thus, our data suggest that, when external sensory input is highly similar in different subregions of an environment, the path integrator fails to drive separation of the patterns and the activity dynamics of the attractor result in highly similar responses in each compartment. Experiments in environments with a higher degree of repetition (e.g., Alvernhe et al. 2008; Singer et al. 2010) have also found that about 50% or more cells discriminated. In such studies, visible extramaze cues were present that could have supported pattern separation. These findings do not therefore stand in strong support of the notion that path integration alone could supply the metric information that could be used to disambiguate otherwise visually identical environments. Our findings instead suggest the opposite—that path integration is not used, or at least not spontaneously. This finding is consistent with recent evidence that visual information can dominate inputs to the hippocampus, such that virtual motion along a cue rich linear track is sufficient to drive spatially localized place cell activity (Chen et al. 2013; Ravassard et al. 2013).

In our study, additional support for the absence of a path integration input was provided by 2 manipulations that were intended to induce remapping of the place cell pattern in one part of the environment. If the global place cell pattern is linked path-integration-wise then alteration of the place cell representation of the environment at one part of the maze might cause nonlocal changes in parts of the representation further away. Some support for this has come from the observation of CA3 place cells expressing a small degree of nonlocal remapping to a geometric change in a 4 compartment hairpin maze (Alvernhe et al. 2008). Here, we find strong local remapping consistent with prior studies (e.g., O'Keefe and Burgess 1996; Lenck-Santini et al. 2004). The alterations seen in the geometric change fit predictions from models of place cell function in which place cells are driven strongly by boundary inputs and re-scale fields in response deformation of boundaries (Hartley et al. 2000). However, no evidence of nonlocal remapping in CA1 cells was observed, changes to the representation remained purely local. This is consistent with a previous report by (Paz-Villagran et al. 2004), who found that remapping in one part of an environment did not spread to separate but connected regions. While our study is similar to Paz-Villagran et al. (2004), our data provide a new and important clarification of the properties of hippocampal place cells. In the study by Paz-Villagran et al., rats first learned contextually different environments separately (e.g., black circle vs. white hexagon), then experienced them connected together and finally experienced contextual change to induce remapping. This ensured that different representations were formed in each of the compartments due to distinct local cues. In our experiment, the environment was learned as a whole, and the representations in the compartments were not distinct prior to testing remapping. Thus, our data show that even when an environment is learned as a whole, remapping does not spread across to other unaltered regions. Our study also differs from previous studies looking for nonlocal remapping in that we specifically included a nongeometric remapping condition. Thus, we can conclude that neither geometric nor nongeometric alterations, which induced local remapping, are sufficient to drive nonlocal remapping in CA1 ensembles.

The findings we report stand in contrast to evidence that place cells can discriminate 2 visually identical regions when an additional nearby region is visually distinct (Paz-Villagran et al. 2006). An important difference between our study and the study of Paz-Villagran et al. (2006) is that the visually identical regions were sectors within a circular arena arranged with different orientations within the overall environment (e.g., one sector faced North East, the other North West), whereas our compartments all faced the same direction (e.g., North). Differences in the heading direction on entry into the compartment would likely be provided by the head-direction cell circuit and provide different inputs to the hippocampal ensemble helping drive remapping in the ensembles. Evidence that different orientations can drive greater separation comes from a study by Fuhs et al. (2005). When 2 visually identical compartments faced in opposite directions (e.g., North vs. South) a high proportion of place cells remapped, whereas this was not the case when the compartments both faced the same direction (e.g., both North).

Our data are consistent with the report by Derdikman et al. (2009) that place cells repeat their firing fields across multiple compartments. Derdikman et al. also found that the grid cells repeated their periodic firing pattern and reset it at transition points in the environment, such as the entry into runway segments. Because simultaneously recorded place cell and grid cell populations appear to alter the spatial location of their firing in concert (Fyhn et al. 2007), we predict that grid cells would show a similar resetting on entry into compartments in the setup we used in the current study. If confirmed, this would indicate a potential role for doorways in isolating subcomponents of the environmental representation, which would be particularly interesting given observations that crossing through a doorway induces a memory decrement in human participants (Radvansky and Copeland 2006). It may be that doorways (i.e., access points across barriers) have a privileged role in structuring and segmenting the neural representation of space. Other environmental features may also play an important role. For example, the orienting light source cue card in such experiments also warrants further investigation, since it may play a strong role in driving resetting during ballistic running due to its capacity to act as a distal anchoring landmark (Derdikman et al. 2009). Also, the height of the walls will be important to investigate. Boundary cells (Solstad et al. 2008; Lever et al. 2009) may play a key role in mediating the resetting as suggested by Derdikman et al. (2009). Are small low-walled enclosures, or clear perspex environments, treated the same as high-walled opaque compartments? The work from Derdikman et al. (2009) suggests there is no different between perspex and opaque walls, but the stereotyped ballistic running combined with the wall dividers may play a strong determinant whether the cells treat the space as a unit or a set of fragments.

Our analysis determined that although some cells appeared to show a degree of “rate remapping” of their peak rate as the distance from the peak compartment increased (see Fig. 3), this was not consistent over the population of cells. Rate remapping, first reported by Hayman et al. (2003) and explored in detail by Leutgeb et al. (2005) has been associated with subtle changes to an environment, or task properties (e.g., Ainge et al. 2007; Allen et al. 2012). Our data are not consistent with a role for path integration in providing a functionally useful discrimination cue for place cells in an environment containing more than 2 identical subregions. We also found that extended experience with the environment did not result in increased differentiation of the spatial firing patterns in compartments. This lack of differentiation is consistent with the results of Singer et al. (2010), who found, if anything, an increase in path equivalence with increased experience (though this may be driven by the stereotyped behavior of the rats). However, our results differ from those of Lever et al. (2002), who found that over time during a foraging task CA1 place cells learned to discriminate 2 similar environments (a circle and a square of a similar size). These findings, like the results of Skaggs and McNaughton (1998), may relate to the binary nature of the discrimination to be acquired. In future studies, it will be important to examine whether grid cells also show a similar lack of rate discrimination over compartments, as the place cells do that we report here.

What do our results mean for the animals' conception of the space they were exploring? Did the rats “know” about the different compartments? The rats were not required to perform a behavioral task in the apparatus, and so did not need to attend to compartment location, and so we have no way of determining whether they realized that there were multiple compartments. It may be that had we trained the animals to attend to the relative location of a compartment within the global space, we could have elicited such evidence and would perhaps have seen discrimination form in the place field pattern. Thus, our experiment does not reveal whether the rats knew about the multiple compartments. Nor does it show that place cells cannot use path integration to distinguish the subcompartments, just that they do not do so automatically. It would be interesting if they could be so trained, because the grid cell-resetting phenomenon alluded to earlier ought, in theory, to make such discrimination difficult for the cells, and by implication the rats.

In summary, we report 3 new findings. First, our results provide new evidence that the automatic pattern separation capabilities of place cells have a limited capacity to make use of path integration to disambiguate identical environmental subcompartments of a multicompartment environment. Second, spatial discrimination did not improve with extended experience in the environment. Third, contextual and geometric changes resulted in purely local remapping of place cell representations. Future work exploring grid cell field patterns, goal-directed choice and the extent to which a region is bounded will be important to advance our understanding of how the brain represents the complex large-scale environments that we and other animals inhabit.

## Funding

This work was supported by a Wellcome Advanced Training Fellowship to H.J.S., a grant from the Wellcome Trust to K.J., a grant from the European Commission Framework 7 (Spacebrain) to K.J. and Axona Ltd, and a studentship from the Biotechnology and Biological Sciences Research Council (BBSRC) to E.M. Funding to pay the Open Access publication charges for this article was provided by the Wellcome Trust, UK.
